# Characterization of Transplant Center Decisions to Allocate Kidneys to Candidates With Lower Waiting List Priority

**DOI:** 10.1001/jamanetworkopen.2023.16936

**Published:** 2023-06-05

**Authors:** Kristen L. King, S. Ali Husain, Miko Yu, Joel T. Adler, Jesse Schold, Sumit Mohan

**Affiliations:** 1 Division of Nephrology, Department of Medicine, Vagelos College of Physicians and Surgeons, Columbia University, New York, New York; 2 Columbia University Renal Epidemiology Group, New York, New York; 3 Department of Surgery and Perioperative Care, Dell Medical School, University of Texas at Austin, Austin; 4 Department of Surgery, University of Colorado, Anschutz Medical Campus, Aurora; 5 Department of Epidemiology, School of Public Health, University of Colorado, Anschutz Medical Campus, Aurora; 6 Department of Epidemiology, Mailman School of Public Health, Columbia University, New York, New York

## Abstract

**Importance:**

Allocation of deceased donor kidneys is meant to follow a ranked match-run list of eligible candidates, but transplant centers with a 1-to-1 relationship with their local organ procurement organization have full discretion to decline offers for higher-priority candidates and accept them for lower-ranked candidates at their center.

**Objective:**

To describe the practice and frequency of transplant centers placing deceased donor kidneys with candidates who are not the highest rank at their center according to the allocation algorithm.

**Design, Setting, and Participants:**

This retrospective cohort study used 2015 to 2019 organ offer data from US transplant centers with a 1-to-1 relationship with their local organ procurement organization, following candidates for transplant events from January 2015 to December 2019. Participants were deceased kidney donors with a single match-run and at least 1 kidney transplanted locally and adult, first-time, kidney-only transplant candidates receiving at least 1 offer for a locally transplanted deceased donor kidney. Data were analyzed from March 1, 2022 to March 28, 2023.

**Exposure:**

Demographic and clinical characteristics of donors and recipients.

**Main Outcomes and Measures:**

The outcome of interest was kidney transplantation into the highest-priority candidate (defined as transplanted after zero declines for local candidates in the match-run) vs a lower-ranked candidate.

**Results:**

This study assessed 26 579 organ offers from 3136 donors (median [IQR] age, 38 [25-51] years; 2903 [62%] men) to 4668 recipients. Transplant centers skipped their highest-ranked candidate to place kidneys further down the match-run for 3169 kidneys (68%). These kidneys went to a median (IQR) of the fourth- (third- to eighth-) ranked candidate. Higher kidney donor profile index (KDPI; higher score indicates lower quality) kidneys were less likely to go to the highest-ranked candidate, with 24% of kidneys with KDPI of at least 85% going to the top-ranked candidate vs 44% of KDPI 0% to 20% kidneys. When comparing estimated posttransplant survival (EPTS) scores between the skipped candidates and the ultimate recipients, kidneys were placed with recipients with both better and worse EPTS than the skipped candidates, across all KDPI risk groups.

**Conclusions and Relevance:**

In this cohort study of local kidney allocation at isolated transplant centers, we found that centers frequently skipped their highest-priority candidates to place kidneys further down the allocation prioritization list, often citing organ quality concerns but placing kidneys with recipients with both better and worse EPTS with nearly equal frequency. This occurred with limited transparency and highlights the opportunity to improve the matching and offer algorithm to improve allocation efficiency.

## Key Points

**Question:**

How often do kidney transplant centers skip candidates with the highest priority to place kidneys with recipients with lower-ranked allocation prioritization?

**Findings:**

This cohort study of 26 579 organ offers from 3136 donors to 4668 recipients found that at 11 geographically isolated transplant centers, kidneys were placed further down the match-run than the candidate with the highest priority 68% of the time. Lower-quality kidneys were statistically significantly more likely to be placed with candidates further down the list than higher-quality kidneys.

**Meaning:**

These findings suggest that transplant centers frequently skip over candidates to place kidneys with recipients with lower allocation priority, with limited oversight and transparency.

## Teaser

This cohort study assesses the practice and frequency of transplant centers placing deceased donor kidneys with candidates with lower priority over those with higher priority according to the allocation algorithm.

## Introduction

During allocation of deceased donor kidneys (DDKs) in the United States, an objective prioritization algorithm ranks the patients on the waiting list eligible to receive a given kidney into a match-run that determines the order in which organ offers are extended.^1,2,3^ Transplant centers may decline the offer for their candidate, resulting in the organ being available to the next candidate on the match-run, who may be listed at the same transplant center or a different center.^4,5^

During allocation, donor organs are preferentially offered to local candidates within defined geographic boundaries before candidates farther away, although criteria determining these boundaries have changed over time. This geographic prioritization can result in multiple patients from a small number of transplant centers dominating the top of the match-run, and in some instances of geographically isolated centers with a 1-to-1 relationship with their local organ procurement organization, candidates from that single center comprise the local match-run. When organs are offered to patients at a 1-to-1 center, that center has complete discretion to decline the offer for some patients but accept it for a patient with lower priority on the waiting list. This introduces a subjective element into an otherwise objective allocation system with potential negative consequences for skipped candidates.^5,6^ Out-of-sequence allocation of kidneys is increasing and potentially contributing to disparities in access to transplantation, yet the role of transplant centers in allowing organs to reach patients with lower priority scores has not been sufficiently studied.^7^

This study examined deceased donor kidney match-runs at 11 one-to-one transplant centers without any other centers eligible for their local donor organs from 2015 to 2019. We describe how frequently kidneys were transplanted into the candidate with the highest priority on the waiting list identified by the allocation algorithm vs transplanted at the discretion of the center into a candidate ranked lower on the waiting list.

## Methods

This cohort study was approved by the Columbia University Medical Center Institutional Review Board. Since this retrospective cohort study used deidentified data from a national transplant registry, informed consent could not be obtained, so the requirement was waived. This study is reported following the Strengthening the Reporting of Observational Studies in Epidemiology (STROBE) reporting guideline.

This study used data from the Scientific Registry of Transplant Recipients (SRTR). The SRTR data system includes data on all donors, transplant candidates on waiting lists, and transplant recipients in the US, submitted by the members of the Organ Procurement and Transplantation Network (OPTN). The Health Resources and Services Administration, US Department of Health and Human Services, provides oversight to the activities of the OPTN and SRTR contractors.

We identified all kidney transplant centers with a 1-to-1 relationship with their organ procurement organization (OPO) and no other centers within their donation service area. Using potential transplant recipient match-run data from 2015 to 2019, we identified all match-runs at OPOs associated with 1-to-1 centers where at least 1 kidney was transplanted locally (additional details in eMethods in Supplement 1). SRTR program-specific reports from June 2016 to June 2020 provided annual center-level transplant and waiting list volume.

We compared how frequently transplanted kidneys went to the local candidate ranked highest in the waiting list (placed after zero local offers declined) vs a candidate later in the match-run, over time and by kidney quality. Kidney Donor Profile Index (KDPI) was used to assess quality; scores range from 0% to 100%, and lower scores indicate better donor quality. We examined how frequently kidneys went to the candidate ranked highest among all included transplants at each center and how far down the match-run kidneys went for kidneys not placed with the candidate ranked highest in terms of the recipient’s match sequence position and the proportion of the local match-run list skipped. We categorized transplant centers’ reasons for declining offers into 5 groups (eTable 1 in Supplement 1).

We compared donor characteristics for kidneys transplanted into candidates with highest vs lower rank. Race and ethnicity were included as categorized in the SRTR transplant registry from the data reported by transplant centers at the time of candidate registration and categorized as Asian, Black, other (eg, Pacific Islander or Native American) or multiracial, and White, and ethnicity was categorized as Hispanic or non-Hispanic. Race and ethnicity were included in analysis because a goal of this study was to understand whether there are disparities by race and ethnicity in offer acceptance patterns for these local donors, since race- and ethnicity-based disparities in access to transplantation have been identified in other related studies. Among the subset of recipients who were both listed and transplanted between 2015 and 2019 (candidates whose complete local offer history was available, with follow-up through December 31, 2019), we compared recipients who had never been skipped vs recipients with at least 1 local DDK offer declined before receiving their transplant.

For each declined offer, we calculated the difference in estimated posttransplant survival (EPTS) score between the skipped candidate and the ultimate recipient of the kidney. EPTS scores range from 0% to 100%, with lower scores indicating longer estimated posttransplant survival. We examined distributions of the differences in EPTS score stratified by the reason for decline and donor KDPI.

### Statistical Analysis

Analysis was performed in Stata/MP statistical software version 17 (StataCorp) by K.L.K.. Groups were compared using χ^2^ for categorical data or Wilcoxon rank-sum tests for continuous data. Two-sided α<.05 determined statistical significance. Data were analyzed from March 1, 2022 to March 28, 2023.

Additionally, we conducted a sensitivity analysis because Kidney Allocation System changes were implemented in March 2021 (eMethods in Supplement 1). Using match-run data from March 16, 2021, to December 31, 2021, for the 11 centers included in our study, we calculated how frequently local kidneys were placed with candidates who were the highest ranked vs lower ranked at the same center under the new allocation system. To assess generalizability of our findings to the other transplant centers without an exclusive 1-to-1 relationship with their local OPO, we also examined kidney placement from local donor match-runs at all 231 transplant centers receiving any offers in 2019.

## Results

At 11 one-to-one centers with no other local transplant centers within their donation service area between 2015 and 2019, we identified 26 579 organ offers from 3136 donors (median [IQR] age, 38 [25 to 51] years; 2903 [62%] men) with a single match-run and from whom at least 1 kidney was transplanted locally, resulting in 4668 kidney transplants. Mean center waiting list size at the start of each year across the study period ranged from 160 to 1279 candidates. A total of 26 579 organ offers were made, and the centers performed a median (IQR) of 101 (65 to 175) DDK transplants and 14 (7 to 38) living donor transplants annually. Local donors were used for a median (IQR) of 82% (67% to 91%) of DDK transplants at these centers.

**Figure 1.**
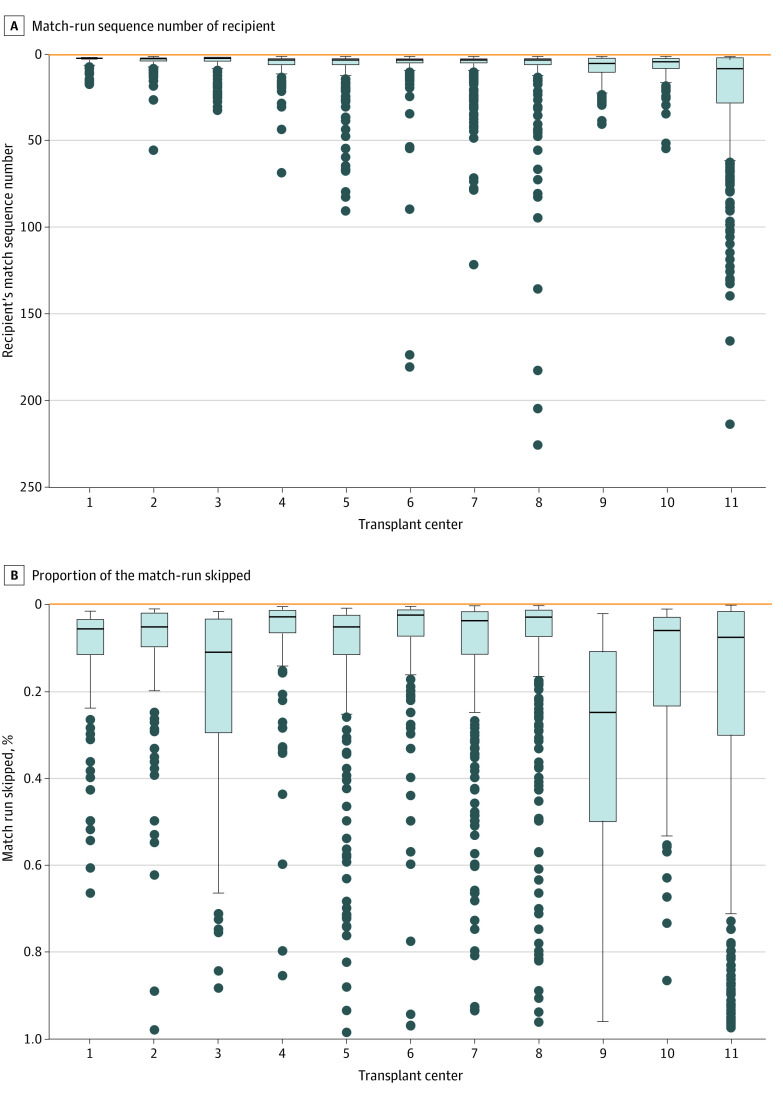
How Far Down the Match-Run Centers Placed Kidneys When Skipping Their Candidate With the Highest Allocation Priority Box and whisker plots depict the allocation prioritization rankings for recipients of transplant events at 11 transplant centers with 1-to-1 relationships with their local organ procurement organization when centers skipped their highest-ranked candidate. Bold lines indicate the median position in the local match run where the recipient was ranked or the median proportion of the match run skipped across all events at that center; boxes, IQR; whiskers, upper and lower adjacent values; and dots, outside values in the distributions at each center.

Among 4668 transplants, 1499 (32%) went to the candidate ranked as their center’s highest priority and 3169 (68%) were placed with a candidate with lower waiting list priority at the same center. Individual centers skipped the candidate ranked highest to place local kidneys with a candidate ranked lower 54% to 77% of the time. Kidneys placed with candidates who were ranked lower went to a median (IQR) of the 4th- (3rd- to 8th-) ranked candidate, with individual centers ranging from placing kidneys at a median (IQR) of the 3rd- (2nd- to 4th-) ranked candidate to the 9th- (4th- to 28th-) ranked candidate (Figure 1A). Centers skipped a median (IQR) of 5.6% (2.1% to 18.4%) of their total match-run to place kidneys further down the list, and all centers skipped at least two-thirds of their match-run at least once (Figure 1B). Among 1532 donors (49%) with both kidneys placed locally with separate recipients, 311 donors (20%) had both kidneys placed with the 2 candidates ranked highest, 291 donors (19%) had 1 kidney placed with the candidate ranked highest and 1 kidney placed after at least 1 declined offer, and 311 donors (61%) had both kidneys placed after at least 1 declined offer.

Higher KDPI (lower quality) kidneys were less likely to be accepted for the candidate ranked highest, with 24% of kidneys with KDPI of at least 85% going to the candidate ranked highest at their center, compared with 44% of kidneys with KDPI of 0% to 20% (eTable 2 in Supplement 1). The reasons provided by centers for declining the offers for candidates ranked higher in the allocation prioritization list were most frequently related to donor age or organ quality (14 332 of 21 911 declined offers [65%]), and less frequently for patient-related (3580 declined offers [16%]) or immunologic (2349 declined offers [11%]) reasons. Donor age or organ quality was cited more frequently as the reason for decline as KPDI increased, accounting for 751 of 1880 declines (40%) for kidneys with KDPI of 0% to 20%, 2028 of 3849 declines (53%) for kidneys with KPDI 21% to 40%, 10 070 of 14 297 declines (70%) for kidneys with KPDI 41% to 84%, and 1483 of 1885 declines (79%) for kidneys with KDPI of 85% or greater (eFigure 1 in Supplement 1).

Kidneys placed with candidates ranked with lower priority came from older donors compared with kidneys accepted for the candidate ranked highest (median [IQR] age, 39 [26 to 52] years vs 35 [24 to 48] years) (Table 1). Kidneys placed lower in the match-run were more frequently from donors with a history of hypertension, smoking, cancer, donation after circulatory death, and blood type O. Kidneys placed lower in the match-run also came more frequently from donors with a biopsy performed (1625 kidneys [51%] vs 576 kidneys [38%]), and were more likely to have findings of glomerulosclerosis greater than 11% in either kidney (346 kidneys [11%] vs 97 kidneys [6%]). Kidneys from Hispanic donors and donors with hepatitis C were more frequently accepted for the candidate ranked highest at their center’s waiting list.

**Table 1.** Donor Characteristics of Deceased Donor Kidneys Transplanted Locally Into the Candidate With the Highest Priority or a Candidate With Lower Allocation Priority at 11 Transplant Centers With a 1-to-1 Relationship With Their Local Organ Procurement Organization From 2015 to 2019

Characteristic	Kidney transplants, No. (%)			*P *value
	All transplanted kidneys (n = 4668)	Recipient’s allocation match-run ranking at their center		
		Top ranked (n = 1499)	Lower ranked (n = 3169)	
Age, median (IQR), y	38 (25-51)	35 (24-48)	39 (26-52)	<.001
Gender				
Women	1765 (38)	542 (36)	1223 (39)	.11
Men	2903 (62)	957 (64)	1946 (61)	
KDPI, %^a^				
Median (IQR)	46 (24-68)	37 (16-60)	52 (27-71)	<.001
KDPI ≥85	340 (7)	83 (6)	257 (8)	.002
Race^b^				
Asian	129 (3)	34 (2)	95 (3)	.09
Black	777 (17)	234 (16)	543 (17)	
Other or multiracial	92 (2)	23 (2)	69 (2)	
White	3670 (79)	1208 (81)	2462 (78)	
Ethnicity				
Hispanic	641 (14)	238 (16)	403 (13)	.003
Non-Hispanic	4027 (86)	1261 (84)	2766 (87)	
Blood type				
O	2191 (47)	655 (44)	1536 (48)	<.001
A	1735 (37)	552 (37)	1183 (37)	
B	595 (13)	225 (15)	370 (12)	
AB	147 (3)	67 (4)	80 (3)	
Diabetes	294 (6)	85 (6)	209 (7)	.23
Hypertension	1246 (27)	340 (23)	906 (29)	<.001
Proteinuria	2037 (44)	630 (42)	1407 (44)	.13
DCD	1077 (23)	274 (18)	803 (25)	<.001
Peak serum creatinine, median (IQR), mg/dL^c^	1.3 (1.0-1.8)	1.3 (1.0-1.7)	1.3 (1.0-1.8)	.37
Biopsy performed	2201 (47)	576 (38)	1625 (51)	<.001
GS ≥11% in either kidney	443 (9)	97 (6)	346 (11)	<.001
Cigarette use >20 pack-years	816 (17)	221 (15)	595 (19)	.001
History of cancer	82 (2)	16 (1)	66 (2)	.01
PHS-IR	918 (20)	309 (21)	609 (19)	.26
HCV (antibody or NAT)	61 (1)	41 (3)	20 (1)	<.001

Abbreviations: DCD, donation after circulatory death; GS, glomerulosclerosis; HCV, hepatitis C virus; KDPI, Kidney Donor Profile Index; NAT, nucleic acid amplification test; PHS-IR, public health service increased risk.

SI conversion factor: To convert creatinine to micromoles per liter, multiply by 88.4.

^a^ Range, 0% to 100%; higher score indicates lower kidney quality.

^b^ The other or multiracial category includes 7 multiracial donors, 27 Native American donors, and 58 Pacific Islander donors.

^c^ Missing creatinine values were excluded for 2 donors (<0.1%).

**Figure 2.**
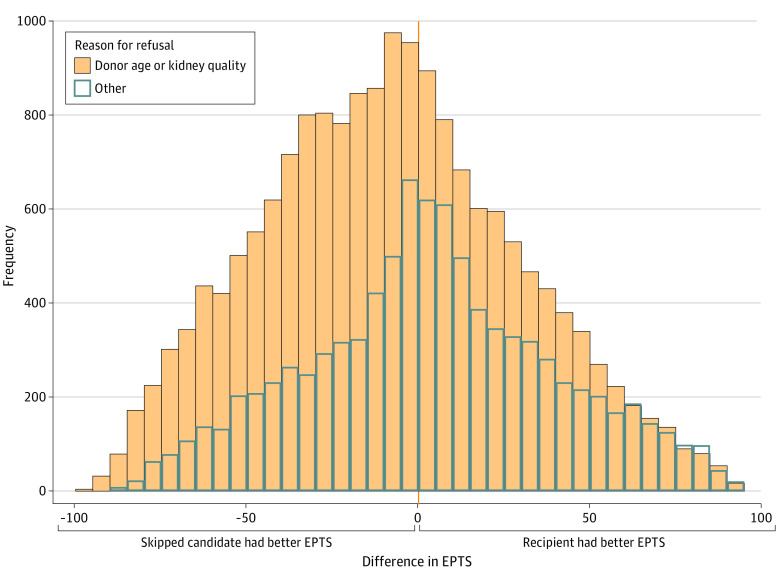
Frequency Histogram of the Difference Between the Estimated Posttransplant Survival (EPTS) Scores of the Skipped Candidates and the Ultimate Recipient for Each Declined Offer, by Reason for Organ Offer Decline

**Figure 3.**
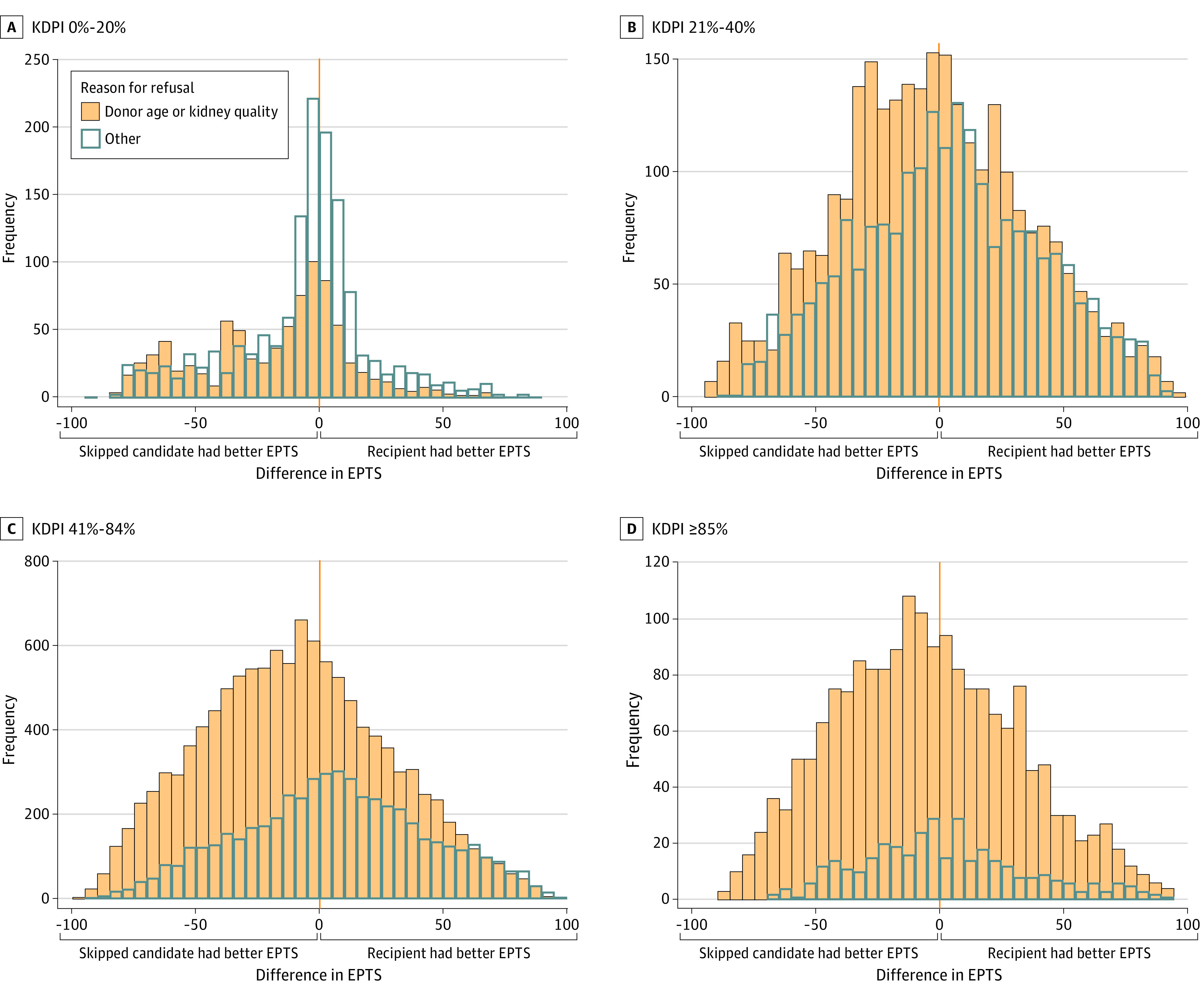
Frequency Histogram of the Difference Between the Estimated Posttransplant Survival (EPTS) Scores of the Skipped Candidates and the Ultimate Recipient for Each Declined Offer, by Reason for Organ Offer Decline and Kidney Donor Profile Index (KDPI)

When comparing the candidate’s EPTS score from each declined offer with the EPTS scores of the eventual recipients, kidneys placed with candidates further down the match-run went to candidates with both better and worse EPTS scores than the skipped candidate (Figure 2). Donor age and quality refusals skewed toward the skipped candidate having better estimated survival than the ultimate recipient (median [IQR] difference, −10 [−36 to 16] points). For all other refusal reasons, the distribution was closer to center, with a skew toward the recipient having better EPTS than the skipped candidate (median [IQR] difference 2 [−21 to 27] points). When stratifying declined offers by donor KDPI, the differences in EPTS between skipped candidates and recipients with lower priority followed similar distributions, with recipients having both better and worse EPTS scores than the skipped candidates (Figure 3; eFigure 2 in Supplement 1). Differences in EPTS were smaller for declined offers of kidneys with KDPI of 0% to 20% (Figure 3A), and there were relatively fewer refusals for reasons other than donor age or kidney quality among kidneys with KDPI of 85% to 100% (Figure 3D).

Among 6024 local candidates added to the waiting list and appearing in match runs between January 1, 2015, and December 31, 2019, 3015 (50%) received a DDK transplant. We compared recipients who were never skipped over for a local kidney (1572 recipients [52%]) vs recipients who were ever skipped before receiving a kidney (1443 recipients [48%]). Never-skipped recipients were more frequently White, Hispanic, and women (Table 2). There were no significant differences by age, end-stage kidney disease (ESKD) cause, diabetes, education, or primary payer. Never-skipped recipients were more likely to be preemptive transplant recipients compared with ever-skipped recipients (302 recipients [19%] vs 233 recipients [16%]; *P* = .03), but among recipients whose transplants were not preemptive, dialysis time was similar between skipped and never-skipped recipients (Table 2). Never-skipped recipients waited longer for their first local kidney offer (median [IQR], 3.7 [1.1 to 9.7] months vs 2.9 [0.9 to 7.5] months) and had better donor quality for their first accepted offer (median [IQR] KDPI, 42%, [20% to 67%] vs 47% [24% to 68%]). Although candidates who were ever skipped had higher KDPI for their first local offer (median [IQR] KDPI, 59% [35% to 75%] vs 42% [20% to 67%]), over the course of their offer history the lowest KDPI offer received was a median (IQR) of 28% (14% to 46%), and they were eventually transplanted with a median (IQR) donor KDPI of 47% (24% to 68%). A similar proportion of never-skipped and ever-skipped recipients were transplanted with kidneys with KDPI of 85% or greater (98 recipients [6%] vs 94 recipients [7%]; *P* = .75). Recipients with obesity were more likely to have been skipped, and among ever-skipped recipients, they were more likely to ever have an offer declined due to donor size or weight (191 of 654 recipients with obesity [29%] vs 134 of 788 recipients without obesity [17%]; *P* < .001).

**Table 2.** Characteristics of Recipients Who Were Never Skipped vs Ever Skipped Before Receiving a Deceased Donor Kidney Transplant, Among Candidates Listed and Transplanted Between 2015 and 2019

Characteristic	Kidney recipients, No. (%)			*P* value
	All recipients (n = 3015)	Never skipped (n = 1572 [52])	Ever skipped (n = 1443 [48])	
Age at listing, median (IQR), y	53 (40-62)	53 (40-62)	53 (41-61)	.62
Race^a^				
Asian	316 (10)	148 (9)	168 (12)	.009
Black	979 (32)	483 (31)	496 (34)	
Other or multiracial	105 (3)	60 (4)	45 (3)	
White	1615 (54)	881 (56)	734 (51)	
Ethnicity				
Hispanic	467 (15)	267 (17)	200 (14)	.02
Non-Hispanic	2548 (85)	1305 (83)	1243 (86)	
Gender				
Men	1900 (63)	957 (61)	943 (65)	.01
Women	1115 (37)	615 (39)	500 (35)	
Blood type				
O	1286 (43)	626 (40)	660 (46)	<.001
A	1185 (39)	601 (38)	584 (40)	
B	360 (12)	242 (15)	118 (8)	
AB	184 (6)	103 (7)	81 (6)	
Cause of ESKD				
Cystic	260 (9)	132 (8)	128 (9)	.44
Diabetes	902 (30)	456 (29)	446 (31)	
Hypertension	580 (19)	294 (19)	286 (20)	
Glomerulonephritis	686 (23)	372 (24)	314 (22)	
Other or unknown	587 (19)	318 (20)	269 (19)	
EPTS score, median (IQR)^b^	36 (13-66)	35 (12-65)	37 (13-66)	.26
BMI at listing				
Median (IQR)^b^	28 (24-32)	28 (24-32)	29 (25-33)	<.001
<30	1738 (58)	950 (60)	788 (55)	.001
≥30	1275 (42)	621 (40)	654 (45)	
History of diabetes^b^	1110 (37)	558 (36)	552 (38)	.11
Education^b^				
≤High school or GED	1347 (45)	733 (47)	614 (43)	.07
Attended college or technical school	779 (26)	398 (25)	381 (27)	
≥Associate’s degree	867 (29)	430 (28)	437 (31)	
Primary payer at listing				
Private	1055 (35)	540 (34)	515 (36)	.14
Medicaid	172 (6)	101 (6)	71 (5)	
Medicare	1733 (57)	897 (57)	836 (58)	
Other	55 (2)	34 (2)	21 (1)	
Preemptively listed	535 (18)	302 (19)	233 (16)	.03
Time receiving dialysis (if not listed preemptively), median (IQR), y	2.2 (1.1-4.1)	2.1 (1.1-4.1)	2.4 (1.1-4.1)	.13
Time from listing to transplant, median (IQR), mo	6.3 (2.0-15.5)	3.7 (1.1-9.8)	10.4 (4.2-22.0)	<.001
Time from first activation to transplant, median (IQR), mo	5.4 (1.7-14.2)	3.2 (1.0-8.7)	9.2 (3.8-20.5)	<.001
Time from listing to first offer, median (IQR), mo	3.3 (1.0-8.6)	3.7 (1.1-9.7)	2.9 (0.9-7.5)	<.001
KDPI^c^				
Median (IQR)	45 (22-67)	42 (20-67)	47 (24-68)	.01
≥85	192 (6)	98 (6)	94 (7)	.75
Lowest KDPI offer received, median (IQR), %	35 (17-57)	42 (20-67)	28 (14-46)	<.001
KDPI of first offer, median (IQR), %	52 (25-72)	42 (20-67)	59 (35-75)	<.001

Abbreviations: BMI, body mass index (calculated as weight in kilograms divided by height in meters squared); ESKD, end stage kidney disease; EPTS, estimated post-transplant survival; KDPI, Kidney Donor Profile Index.

^a^ The other or multiracial category includes 53 multiracial recipients, 17 Pacific Islander recipients, and 35 Native American recipients.

^b^ Participants with missing values excluded: 2 recipients (<0.1%) for BMI, 5 recipients (0.2%) for diabetes, 10 recipients (0.3%) with education (not available due to age), 12 recipients (0.4%) with education data missing; 5 recipients (0.2%) for EPTS.

^c^ Range, 0% to 100%; higher score indicates lower kidney quality.

In the sensitivity analysis, after competition for local kidneys was introduced under the KAS-250 allocation system, the 11 previously isolated centers placed local kidneys with a candidate with lower rank at their center for 208 of 342 transplants (61%) between March 16, 2021, and December 31, 2021. When examining transplant patterns at all 231 transplant centers performing any DDK transplants from local match runs in 2019, we found that kidneys were placed with recipients ranked lower at the same center for 6971 of 9216 transplants (76%). Centers ranged from placing 0% to 100% of their kidneys with candidates with lower allocation priority, although the centers at these 2 extremes tended to be lower-volume centers (eFigure 3 in Supplement 1).

## Discussion

In this cohort study, we found that lower-quality organs were more likely to be declined for the candidate ranked highest and accepted for patients with lower priority, presumably hoping that shorter wait times for candidates with lower priority would offset the lower organ quality. However, we also found that many high-quality organs were declined for candidates ranked highest, reportedly due to organ quality concerns, but they were deemed acceptable for candidates with lower priority with longer survival prospects. Even among the highest-quality kidneys (ie, KDPI, 0%-20%), only 44% were placed with the candidate with the highest allocation priority points, demonstrating that transplant centers often overlook higher allocation priority candidates to place organs with the “right” recipient. As a result, candidates with lower priority were often transplanted with kidneys declined for higher priority candidates with both better and worse relative estimated survival. These findings provide context for why the number of declined offers an organ accumulates is not an independent reflection of its quality.^8^ This suggests that centers may be engaging in their own organ-recipient longevity matching that is more nuanced than the current EPTS-KDPI matching system, or using other factors to determine which patient should receive a given organ. Concerns that the current allocation algorithm provides inadequate specificity of the candidates who are most likely to benefit from a given organ may inadvertently create the sense that centers must do more to match organs to the “right patient,” thus encouraging list diving, ie, the observed behavior of placing organs with candidates with lower priority according to the official allocation algorithm, especially for the highest-quality organs.

Kidney transplantation is the best treatment for ESKD with better survival, quality of life, and cost compared with dialysis.^9,10,11,12,13^ With a donor kidney shortage, patients typically spend several years on the waiting list before receiving a DDK transplant, and ensuring fair allocation of donated kidneys remains a priority.^14^ Although the allocation system provides an objective prioritization of matched candidates for each kidney, most kidneys are placed with a candidate who is not the highest priority according to the allocation algorithm.^4,5,8^ Given that more than 99% of DDK offers are declined, frequently without the intended recipient’s knowledge,^4^ these declined offers are presumably rooted in centers’ belief that patients will receive better quality organ offers within a reasonable timeframe, especially if they have accrued sufficient priority points to reach the top of the match-run.^5,15^

Notably, 1-to-1 centers know they can skip candidates at their center—and in some instances, nearly the entire waiting list—without losing the kidney to another center, which essentially allows them to select any specific recipient regardless of official allocation prioritization. This is of particular concern, given prior analyses demonstrating the subjective nature of organ offer declines and differences based on recipient race and comorbidities, including obesity.^4,16^ Understanding how organ placement deviates in practice from the official allocation prioritization scheme can help identify implicit biases in organ acceptance, inefficiencies in allocation (perceived and actual) and opportunities for improvements in future iterations.^17,18,19^ While patients may also decline an organ or be temporarily unsuitable for transplantation at the time of an offer, we found that patient-related refusal codes only accounted for 14% of the declines.

While our analysis primarily focused on centers without local competition and therefore with greater ability to direct organs to predetermined candidates, this is a widespread behavior that occurs at nearly all centers but has not been previously characterized, to our knowledge. These declined offers may contribute to increased cold ischemia for these organs, with attendant concerns about organ viability and increasing discard of kidneys.^19,20,21,22,23^ The observed increased use of kidneys from donors with hepatitis C for the candidate ranked highest suggests that the specific consent process for these kidneys facilitates more expeditious allocation of these organs by limiting the match-run to patients specifically identified by centers as both interested and likely to benefit from these organs. This is an example of how better-tailored allocation using specific donor characteristics coupled with more emphasis on patient preferences could help.^1,15,24,25^

Overlooking candidates in favor of patients lower in the priority ranking can have negative implications for the skipped candidates.^15^ While these candidates may receive a future offer that will be accepted, death and delisting without transplantation are common outcomes for skipped patients.^5^ These transplant center practices, coupled with OPO behavior that uses out-of-sequence organ placement to allocate kidneys to predetermined transplant centers with little accountability, undermine the intended objective design of the allocation system in a manner that is shrouded from both patients and regulatory oversight and risks undermining the trust that patients and donor families have in a fair and equitable system.^7^ Additionally, new ESKD value-based care models in the United States place a significant premium on kidney transplantation, thus allowing transplant centers to potentially influence reimbursements, performance, and efficacy of these payment models.^26^

To our knowledge, our analysis is the first quantitative description of the phenomenon of list diving. As shown in our sensitivity analysis, this practice is not limited to 1-to-1 transplant centers and persists despite recent changes in allocation leading to broader organ sharing and fewer single-center OPOs. List diving appears to continue unabated and could even be increasing under the newest allocation system, highlighting the need to better understand and potentially mitigate this phenomenon. Future allocation policies need to take into consideration potential recipients’ characteristics and preferences in a manner that results in match runs that are viewed by clinicians as providing more appropriate organ offers for their patients. This would ideally lower or eliminate declined offers (and out-of-sequence offers) by reducing or eliminating inappropriate offers for given recipients, thus dramatically improving allocation efficiency and organ placement in a patient-centered manner.^7,27^ Increased transparency for declined offers is an essential guardrail to ensure that the allocation system remains objective, accountable, and patient-centered and does not inadvertently increase disparities in access to transplantation.^5,8,15,28^

### Limitations

This study has some limitations. Despite KDPI and EPTS being imperfect surrogate measures of organ quality and patient longevity, respectively, they are currently used as part of the kidney allocation system, but the extent to which they are considered in clinical organ offer decisions may vary among centers. Further study is needed to understand how allocation behavior may have changed at all centers under the new Kidney Allocation System as more data become available.

## Conclusions

In this cohort study of local kidney allocation at isolated transplant centers, we found that centers frequently skipped their candidates with the highest priority to place kidneys with recipients further down the allocation prioritization list, often citing organ quality concerns but placing kidneys with recipients with both better and worse EPTS with nearly equal frequency. Given the dramatic effect that kidney transplantation has on quality of life and survival, patients have a right to information about factors that influence their ability to receive a transplant, especially potentially modifiable factors, such as center preferences and behavior or perceptions of patient preferences. The common nature of skipping patients with the highest priority suggests that improvements to the allocation algorithms are needed to better incorporate the needs and preferences of patients and more precisely match specific organs to recipients. Improving allocation efficiency would reduce organ offer declines and the need for out-of-sequence placements by OPOs or patient-skipping by transplant centers, which could potentially reduce the discard of viable kidneys.^7^ Our findings emphasize the need for improving allocation system efficiency, improving transparency, and establishing process measures for monitoring the transplant system in the next iteration of deceased donor kidney allocation.

## Notes

Supplement 1.
eMethods.
**eTable 1.** Categorizing Reasons Provided by Transplant Centers for Declining Local Deceased Donor Kidney Offers at Transplant Centers With a 1-to-1 Relationship With Their Organ Procurement Organization, 2015-2019 **eTable 2.** Frequency of Kidneys Transplanted Into the Candidate With the Highest Priority in the Match-Run vs a Candidate Further Down the Match-Run Among Local Deceased Donor Kidney Transplants at 11 Transplant Centers With a 1-to-1 Relationship With Their Organ Procurement Organization, by Match-Run Year and Kidney Donor Profile Index (KDPI) **eFigure 1.** Reasons for Declining the Deceased Donor Kidney Offer, by Kidney Donor Profile Index (KDPI) **eFigure 2.** Density Histograms of the Difference Between the Estimated Posttransplant Survival (EPTS) Scores of the Skipped Candidates and the Ultimate Recipient for Each Declined Offer, by Reason for Organ Offer Decline and Kidney Donor Profile Index (KDPI) **eFigure 3.** Center-Level Local Deceased Donor Kidney Transplant Volume in 2019 vs the Proportion of Local Transplants That Went to a Recipient Ranked Lower Among 231 Kidney Transplant Centers

Supplement 2.
Data Sharing Statement
