# Supplementary material for: Characterization of Transplant Center Decisions to Allocate Kidneys to Candidates With Lower Waiting List Priority
Source: JAMA Netw Open. 2023 Jun 5;6(6):e2316936. doi: 10.1001/jamanetworkopen.2023.16936 (PMC10242426; doi:10.1001/jamanetworkopen.2023.16936)
Supplement: Supplement 1. — eMethods. eTable 1. Categorizing Reasons Provided by Transplant Centers for Declining Local Deceased Donor Kidney Offers at Transplant Centers With a 1-to-1 Relationship With Their Organ Procurement Organization, 2015-2019 eTable 2. Frequency of Kidneys Transplanted Into the Candidate With the Highest Priority in the Match-Run vs a Candidate Further Down the Match-Run Among Local Deceased Donor Kidney Transplants at 11 Transplant Centers With a 1-to-1 Relationship With Their Organ Procurement Organization, by Match-Run Year and Kidney Donor Profile Index (KDPI) eFigure 1. Reasons for Declining the Deceased Donor Kidney Offer, by Kidney Donor Profile Index (KDPI) eFigure 2. Density Histograms of the Difference Between the Estimated Posttransplant Survival (EPTS) Scores of the Skipped Candidates and the Ultimate Recipient for Each Declined Offer, by Reason for Organ Offer Decline and Kidney Donor Profile Index (KDPI) eFigure 3. Center-Level Local Deceased Donor Kidney Transplant Volume in 2019 vs the Proportion of Local Transplants That Went to a Recipient Ranked Lower Among 231 Kidney Transplant Centers [file jamanetwopen-e2316936-s001.pdf]

Supplemental Online Content

King KL, Husain SA, Yu M, Adler JT, Schold J, Mohan S. Characterization of transplant center decisions to allocate kidneys to candidates with lower waiting list priority. *JAMA Netw Open*. 2023;6(6):e2316936. doi:10.1001/jamanetworkopen.2023.16936

eMethods.

eTable 1. Categorizing Reasons Provided by Transplant Centers for Declining Local Deceased Donor Kidney Offers at Transplant Centers With a 1-to-1 Relationship With Their Organ Procurement Organization, 2015-2019

eTable 2. Frequency of Kidneys Transplanted Into the Candidate With the Highest Priority in the Match-Run vs a Candidate Further Down the Match-Run Among Local Deceased Donor Kidney Transplants at 11 Transplant Centers With a 1-to-1 Relationship With Their Organ Procurement Organization, by Match-Run Year and Kidney Donor Profile Index (KDPI)

eFigure 1. Reasons for Declining the Deceased Donor Kidney Offer, by Kidney Donor Profile Index (KDPI)

eFigure 2. Density Histograms of the Difference Between the Estimated Posttransplant Survival (EPTS) Scores of the Skipped Candidates and the Ultimate Recipient for Each Declined Offer, by Reason for Organ Offer Decline and Kidney Donor Profile Index (KDPI)

eFigure 3. Center-Level Local Deceased Donor Kidney Transplant Volume in 2019 vs the Proportion of Local Transplants That Went to a Recipient Ranked Lower Among 231 Kidney Transplant Centers

This supplemental material has been provided by the authors to give readers additional information about their work.

eMethods

Data Cleaning

Using potential transplant recipient (PTR) match-run data from 2015-2019, we identified all match-runs local to the OPOs associated with OTO centers (n=5984). We excluded donors with zero kidneys placed locally (n=2545) or multiple match-runs generated (n=303), and dropped bypassed offers, offers past the final local acceptance, and offers to non-local candidates (e.g., rare, national 0-ABDR mismatch or high cPRA candidates that top the match-run). Gender and race/ethnicity were included as classified in the SRTR database. Data on donor characteristics were nearly complete (>98% complete for all variables). Missing or unknown values were combined into a "No/Unknown" category for the following donor variables: donor history of diabetes (n = 15, 0.32% unknown), donor history of hypertension (n = 27, 0.58% missing), donor proteinuria (n = 37, 0.79% unknown), donor history of cancer (n = 19, 0.41% unknown), donor history of smoking (n = 85, 1.8% unknown). For missing continuous values, complete case analysis was performed with the excluded missing values noted in the table footnotes: donor creatinine (n = 2, 0.04% missing). For recipient data, all variables were >99% complete and complete case analysis was performed with the exclusions of missing values noted in the table footnotes. Preemptive listing was defined as no dialysis start date reported or a dialysis start date later than the initial listing date.

EPTS Comparisons

For each declined offer, we calculated the difference in Estimated Post-Transplant Survival (EPTS) score between the skipped candidate and the ultimate recipient of the kidney. If both donor kidneys were transplanted, although the skipped candidates would appear only once in the match-run, they were compared against the recipient of any kidney they were eligible to receive. For example, if the kidneys were accepted for the candidates at match sequence positions 2 and 4, the skipped candidate ranked at number 1 would be compared to both recipients at 2 and 4, and the skipped candidate ranked at number 3 would be compared to the recipient at 4 only.

Sensitivity Analyses

Significant changes to the Kidney Allocation System were implemented in March 2021 that eliminated local Donation Service Area (DSA) boundaries and incorporated distance from the donor hospital in the allocation prioritization algorithm in an effort to improve geographic equity in kidney transplantation. Under this new distance-based allocation system (referred to as "KAS-250"), the 11 transplant centers included in our study that had previously experienced a one-to-one relationship with their Organ Procurement Organization (OPO) would now be more likely to have additional candidates listed at other transplant centers appearing in the match runs for the local donors recovered by their OPO. As a result, if the transplant center did not accept the organ offer for their highest-ranked candidate, they now had a greater chance of losing that kidney to a candidate at another center before it could be allocated to the next-ranked candidate at their own center. To examine whether the observed patterns in kidney placement at these centers held under a new allocation system with more competition from other transplant centers, we repeated our primary analysis using the data that was available from the first 9 months of the new allocation system (3/16/2021-12/31/2021).

© 2023 King KL et al. *JAMA Network Open.*

To help contextualize the findings at these 11 centers, we also examined kidney placement patterns at all 231 transplant centers receiving any deceased donor kidney offers in 2019. We calculated how frequently transplant centers placed kidneys from their local donor match runs with their highest-ranked candidate versus a lower-ranked candidate at their center, both overall and by individual center.

© 2023 King KL et al. *JAMA Network Open.*

eTable 1. Categorizing Reasons Provided by Transplant Centers for Declining Local Deceased Donor Kidney Offers at Transplant Centers With a 1-to-1 Relationship With Their Organ Procurement Organization, 2015-2019

Category	Code	Reason	N	%	Total
Patient-Related	800	Patient's condition improved, transplant not needed	9	0.04	
	801	Patient ill, unavailable, refused, or temporarily unsuitable	3,045	13.9	
	802	Multiple organ transplant or different laterality is required	406	1.85	
	803	Patient transplanted	120	0.55	16.34
Organ/Donor Quality	830	Donor age or quality	11,464	52.32	
	831	Donor size/weight	2,415	11.02	
	833	Donor social history	138	0.63	
	834	Positive serological tests	43	0.2	
	835	Organ preservation	87	0.4	
	836	Organ anatomical damage or defect	17	0.08	
	837	Organ-specific donor issue	168	0.77	65.42
Logistical	820	Heavy workload	1	0	
	822	Exceeded one hour response time	4	0.02	
	823	Surgeon unavailable	1	0	
	824	Distance to travel or ship	232	1.06	
	825	Operational - transplant center	27	0.12	1.20
Immunologic	810	Positive crossmatch	605	2.76	
	811	Number of HLA mismatches unacceptable	88	0.4	
	812	No serum	214	0.98	
	813	Unacceptable Antigens	587	2.68	
	815	High CPRA	855	3.9	10.72
Other	898	Other	810	3.7	
	832	Donor ABO	5	0.02	
	860	Medical urgency of another potential recipient	46	0.21	
	880	Kidney placed with extra-renal	358	1.63	
	.	Missing	11	0.05	
	2000	Code not found in data dictionary	154	0.7	
	2001	Code not found in data dictionary	1	0	6.31

© 2023 King KL et al. *JAMA Network Open.*

eTable 2. Frequency of Kidneys Transplanted Into the Candidate With the Highest Priority in the Match-Run vs a Candidate Further Down the Match-Run Among Local Deceased Donor Kidney Transplants at 11 Transplant Centers With a 1-to-1 Relationship With Their Organ Procurement Organization, by Match-Run Year and Kidney Donor Profile Index (KDPI)

	**All Transplants (col %)** n = 4,668	**Recipient was the Top-Ranked Candidate, N (row %)** n = 1,499 (32)	**Recipient was Further Down the Match Run, N (row %)** n = 3,169 (68)
**Match Year**			
2015	907 (19)	276 (30)	631 (70)
2016	982 (21)	260 (26)	722 (74)
2017	965 (21)	322 (33)	643 (67)
2018	918 (20)	327 (36)	591 (64)
2019	896 (19)	313 (35)	582 (65)
**Donor KDPI**			
0-20	1,016 (22)	451 (44)	565 (56)
21-40	1,068 (23)	371 (35)	697 (65)
41-84	2,244 (48)	594 (26)	1,650 (74)
85-100	340 (7)	83 (24)	257 (76)

© 2023 King KL et al. *JAMA Network Open.*

eFigure 1. Reasons for Declining the Deceased Donor Kidney Offer, by Kidney Donor Profile Index (KDPI)

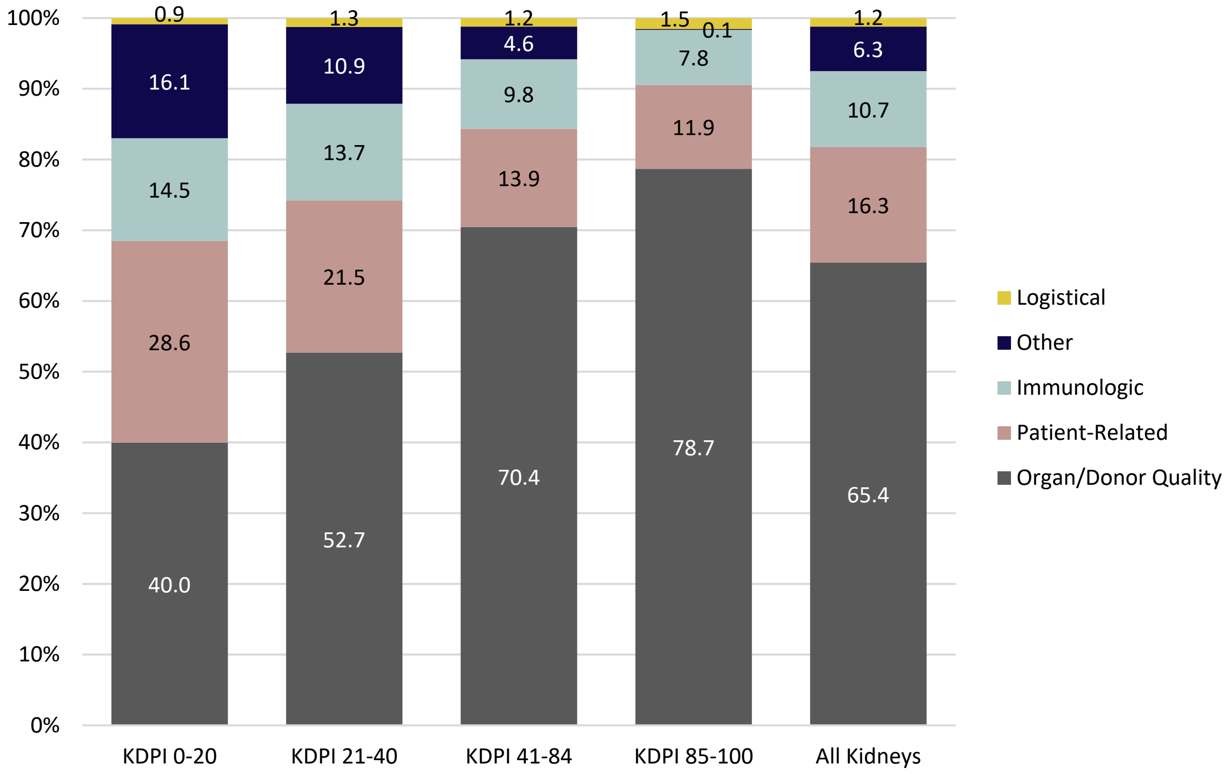

© 2023 King KL et al. *JAMA Network Open.*

eFigure 2. Density Histograms of the Difference Between the Estimated Posttransplant Survival (EPTS) Scores of the Skipped Candidates and the Ultimate Recipient for Each Declined Offer, by Reason for Organ Offer Decline and Kidney Donor Profile Index (KDPI)

A negative difference indicates the candidate who was skipped over had better estimated survival compared to the ultimate recipient, and a positive difference indicates the recipient had better estimated survival compared to the skipped candidate.

A) KDPI 0-20

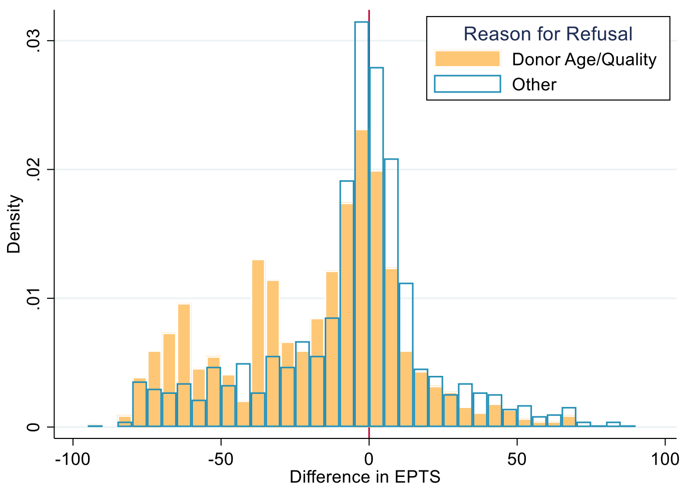

B) KDPI 21-40

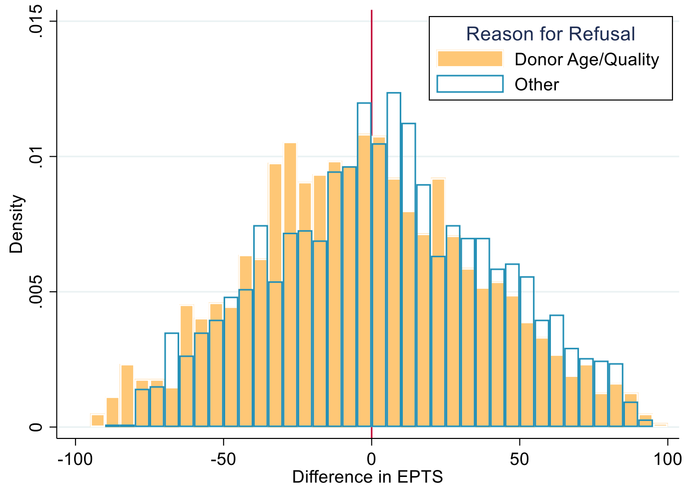

C) KDPI 41-84

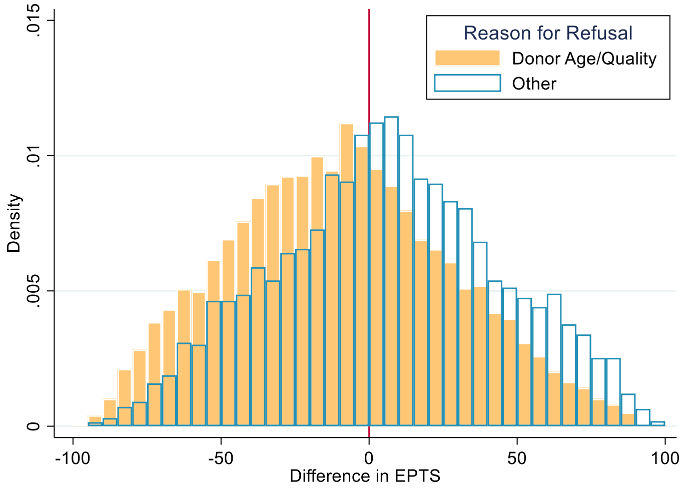

D) KDPI 85-100

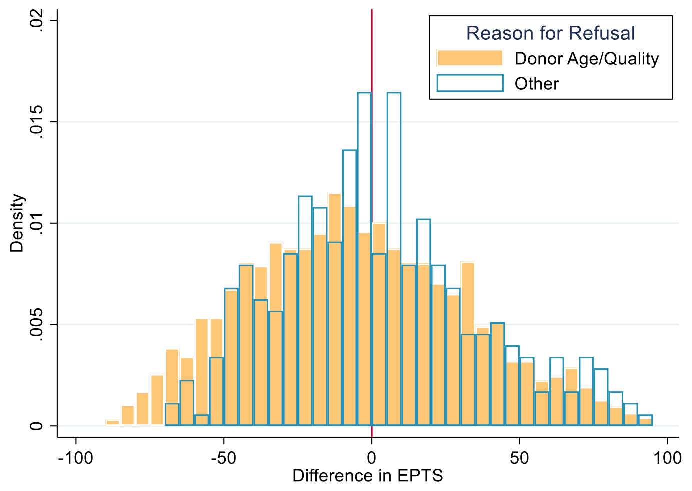

© 2023 King KL et al. *JAMA Network Open.*

eFigure 3. Center-Level Local Deceased Donor Kidney Transplant Volume in 2019 vs the Proportion of Local Transplants That Went to a Recipient Ranked Lower Among 231 Kidney Transplant Centers

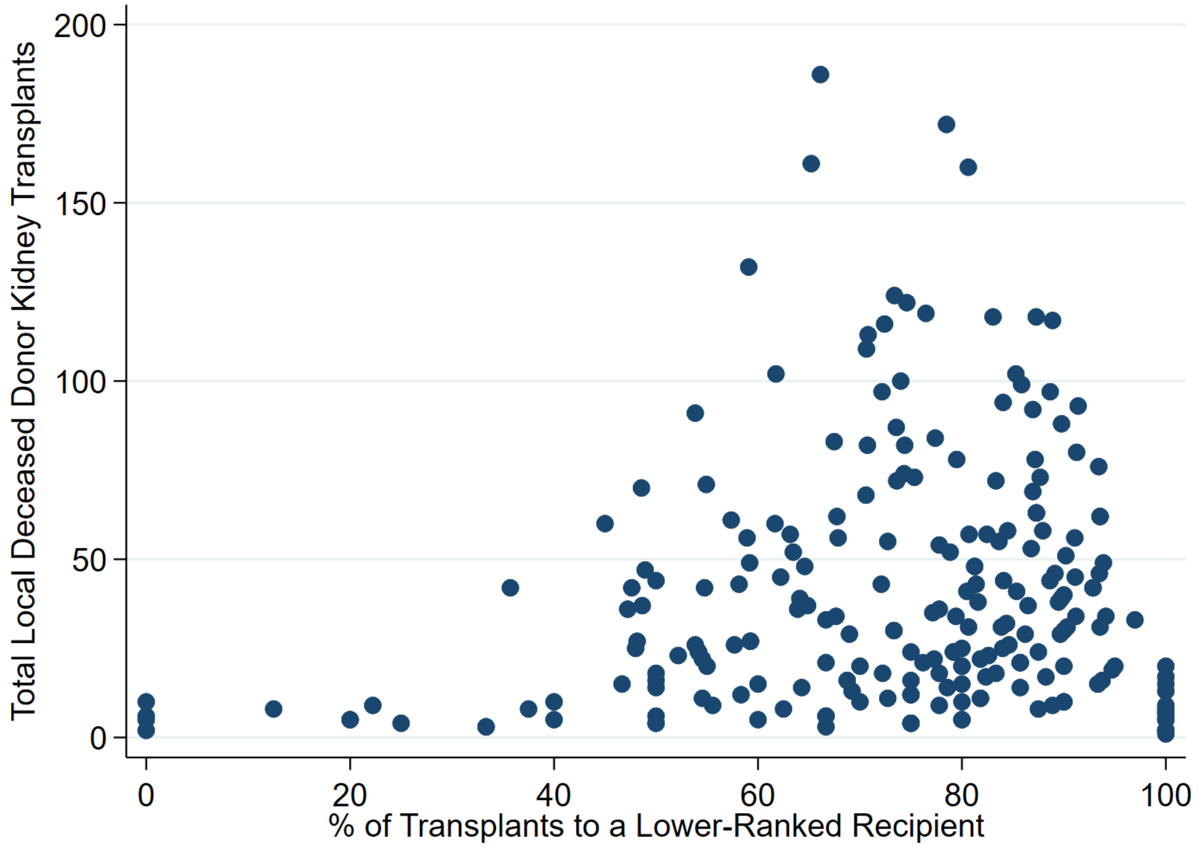

© 2023 King KL et al. *JAMA Network Open.*
