# Supplementary material for: Characterization of Transplant Center Decisions to Allocate Kidneys to Candidates With Lower Waiting List Priority
Source: JAMA Netw Open. 2023 Jun 5;6(6):e2316936. doi: 10.1001/jamanetworkopen.2023.16936 (PMC10242426; doi:10.1001/jamanetworkopen.2023.16936)
Supplement: Supplement 2. — Data Sharing Statement [file jamanetwopen-e2316936-s002.pdf]

Data Sharing Statement

King. Characterization of Transplant Center Decisions to Allocate Kidneys to Candidates With Lower Waiting List Priority. *JAMA Netw Open*. Published June 05, 2023.
doi:10.1001/jamanetworkopen.2023.16936

Data
Data available: No

Additional Information
Explanation for why data not available: Data are available from the Scientific Registry of Transplant Recipients (SRTR) upon request and completion of a research plan and data use agreement.
